# DIR-visible grey matter lesions and atrophy in multiple sclerosis: partners in crime?

**DOI:** 10.1136/jnnp-2014-310142

**Published:** 2015-04-29

**Authors:** Steven H P van de Pavert, Nils Muhlert, Varun Sethi, Claudia A M Wheeler-Kingshott, Gerard R Ridgway, Jeroen J G Geurts, Maria Ron, Tarek A Yousry, Alan J Thompson, David H Miller, Declan T Chard, Olga Ciccarelli

**Affiliations:** 1 NMR Research Unit, Queen Square Multiple Sclerosis Centre, UCL Institute of Neurology, London, UK; 2 School of Psychology and Cardiff University Brain Research Imaging Centre, Cardiff University, Cardiff, Glamorgan, UK; 3 Wellcome Trust Centre for Neuroimaging, UCL Institute of Neurology, London, UK; 4 Nuffield Department of Clinical Neurosciences, FMRIB Centre, University of Oxford, Oxford, UK; 5 Department Anatomy & Neurosciences, Section of Clinical Neuroscience, VU University Medical Centre, Amsterdam, The Netherlands; 6 Sara Koe PSP Research Centre, UCL Institute of Neurology, London, UK; 7 NIHR UCL/UCLH Biomedical Research Centre, London, UK

**Keywords:** MULTIPLE SCLEROSIS, MRI, IMAGE ANALYSIS, PATHOLOGY

## Abstract

**Background:**

The extent and clinical relevance of grey matter (GM) pathology in multiple sclerosis (MS) are increasingly recognised. GM pathology may present as focal lesions, which can be visualised using double inversion recovery (DIR) MRI, or as diffuse pathology, which can manifest as atrophy. It is, however, unclear whether the diffuse atrophy centres on focal lesions. This study aimed to determine if GM lesions and GM atrophy colocalise, and to assess their independent relationship with motor and cognitive deficits in MS.

**Methods:**

Eighty people with MS and 30 healthy controls underwent brain volumetric T1-weighted and DIR MRI at 3 T, and had a comprehensive neurological and cognitive assessment. Probability mapping of GM lesions marked on the DIR scans and voxel- based morphometry (assessing GM atrophy) were carried out. The associations of GM lesion load and GM volume with clinical scores were tested.

**Results:**

DIR-visible GM lesions were most commonly found in the right cerebellum and most apparent in patients with primary progressive MS. Deep GM structures appeared largely free from lesions, but showed considerable atrophy, particularly in the thalamus, caudate, pallidum and putamen, and this was most apparent in secondary progressive patients with MS. Very little co-localisation of GM atrophy and lesions was seen, and this was generally confined to the cerebellum and postcentral gyrus. In both regions, GM lesions and volume independently correlated with physical disability and cognitive performance.

**Conclusions:**

DIR-detectable GM lesions and GM atrophy do not significantly overlap in the brain but, when they do, they independently contribute to clinical disability.

## Introduction

Grey matter (GM) pathology has emerged as a significant and clinically relevant component of multiple sclerosis (MS). Advances in MRI technology have allowed the assessment of GM pathology in MS in vivo. Both GM volume loss (atrophy) and GM lesions occur in MS, and both correlate with neurological and cognitive deficits.^1^ However, a key question is whether they share a common pathogenic mechanism, in particular whether lesions are the cause of atrophy or are caused by an independent process that contributes to clinical outcomes.

Histopathological studies have identified extensive cortical demyelination in people with MS and in those with progressive disease, GM lesion volume may exceed that of WM lesions.^2^ However, it has proven difficult to detect in vivo GM lesions using conventional MRI techniques. Only a small percentage of GM lesions are identified on T2-weighted (<10%) and on fluid-attenuated inversion recovery (FLAIR) scans.^3^ The development of double inversion recovery (DIR) MRI has nearly doubled the detection of GM lesions.^4^ DIR studies demonstrate that GM lesions are spread throughout the cortex, appear early in the course of the disease and accumulate over time.^5^ ^6^

Deep and cortical GM atrophy is now well-recognised in MS and appears to accelerate as patients with MS enter a progressive phase of the disease.^1^ GM atrophy is thought to mark irreversible tissue loss and it is likely to reflect a combination of neuronal morphological changes and loss, and glial abnormalities.^1^ Previous in vivo work provides insight into the mechanisms of GM injury in MS. Two studies have assessed the relationship between GM atrophy and WM injury, and these showed that lesions in connecting WM tracts are associated with deep GM atrophy.^7^ ^8^ Lesions could directly cause localised atrophy, as suggested by pathological studies showing axonal transection and loss in cortical lesions.^1^ There has been little histopathological work examining the co-localisation of cortical atrophy and demyelination, but in the only study we are aware of, local cortical thickness did not correlate with demyelination.^9^ However, fixation can affect cortical thickness (and fixation time may be correlated with it^9^), and so it is possible that this may have obscured an association. As such, it is preferable to look for associations in fresh tissue samples or, better still, in vivo.

This study sought to clarify the spatial overlap between GM atrophy and GM lesions, as well as their independent relationship with cognitive and physical disability, in a large cohort of patients with MS and in different MS subtypes.

## Methods

We recruited 80 people who fulfil the following inclusion criteria: diagnosis of clinically definite MS;^10^ absence of a relapse or use of corticosteroids within the preceding 4 weeks; age <65 years; and no other neurological conditions which could have influenced the pattern of GM atrophy. Thirty healthy volunteers with no known neurological disease were also studied. Patients were consecutively recruited from the National Hospital's specialist MS clinics. Status of disease modifying therapy (DMT), antidepressant for either depression or neuropathic pain, and benzodiazepine for spasms or insomnia were recorded. All participants gave written informed consent.

Clinical status was assessed using the Expanded Disability Status Scale (EDSS)^11^ and the MS functional composite score (MSFC) which includes walking speed on the 25-foot Timed Walk Test (25TWT), 9-hole Peg Test (9HPT) and the Paced Auditory Serial Addition Test (PASAT). Z-scores of these tests were calculated using published means and SDs. Levels of anxiety and depression were also assessed using the Hospital Anxiety and Depression Scale.^12^

All subjects underwent cognitive testing to assess executive function and memory. Executive function was assessed using the Hayling Sentence Completion Task^13^ and Stroop Task,^14^ from which averaged z-scores were calculated on the basis of healthy control performance, and the Symbol Digit Modalities Test (SDMT), from which the age-adjusted z-scores were calculated based on published normative values.^15^ Memory function was evaluated using a composite score of story recall (immediate and 30 min delay) and figure recall (immediate and 30 min delay) from the Adult Memory and Information Processing Battery,^16^ and word and face recognition from the Recognition Memory Test;^17^ z-scores based on the performance of the control sample were also calculated for these tests. When participants were not able to complete the task, their missing data would not be considered for the task's analysis; these were executive function in one patient with primary progressive MS (PPMS), 25TWT in 3 PPMS, 1 relapsing–remitting MS (RRMS) and 9 patients with secondary progressive MS (SPMS), and 9HPT in 1 RRMS and 1 patient with SPMS.

### MRI protocol

Brain MRI was performed on a Phillips 3 T Achieva TX system (Philips Healthcare, Best, the Netherlands) using a 32-channel receive-only coil. T1-weighted (TR=6.9 ms, TE=3.1 ms, TI=824.5 ms, SENSE=2, voxel size=1×1×1 mm), turbo FLAIR (TR=8000 ms, TE=125 ms, TI=2400 ms, SENSE=1.3, voxel size=1×1×3 mm), and DIR (TR=16 000 ms, TE=9.9 ms, TI=2400/325 ms, SENSE=4.16, 1×1×3 mm) sequences were acquired.

### Image registration and lesion mapping

To limit the impact of WM lesions on tissues segmentations, T1-weighted (T1w) hypointense lesions were filled^18^ and the lesion-filled T1w images were then segmented using SPM8 (http://www.fil.ion.ucl.ac.uk/spm/). Anatomical normalisation to Montreal Neurological Institute (MNI) space was achieved via a custom diffeomorphic anatomical registration through exponentiated Lie algebra (DARTEL) template,^19^ generated from all subjects’ (N=110) GM tissue segmentations. To make use of MNI atlases, the DARTEL GM template was affine-registered to the MNI standard space, and each subject's T1-weighted GM was first non-linearly registered to the DARTEL template, and subsequently affine registered to MNI using the DARTEL to MNI transformation, moving it into MNI template space. This pipeline reduces the adverse effect of disease-associated brain atrophy on the registration accuracy. Spatially normalised T1-weighted images were averaged over subjects to form a study-specific T1-weighted MNI-space template. When spatially normalising the segmented GM images to MNI space (via the DARTEL and affine transformations), the images were modulated by the Jacobian determinants so that intensities represent the amount of deformation needed to normalise the images. All registrations were reviewed by SvdP to confirm their accuracy. Modulated, normalised, segmented GM images were smoothed with an 8 mm full-width at half-maximum Gaussian kernel.^20^

GM lesions were marked on the DIR scans using JIM (V.6.0, Xinapse Systems, Northants, UK) by two independent raters (VS and SvdP) according to consensus guidelines.^21^ Marking of lesions was compared between raters and a consensus was reached on all lesions. Subsequently, one in five scans were analysed by DC, after which a final consensus was reached. Total GM lesion volume was then calculated.

Each participant's DIR scan was affine registered to their T1-weighted scan; their DIR-to-T1 affine transformation, T1-to-DARTEL deformation field and DARTEL-to-MNI affine transformation were combined and used to move the binarised GM lesion mask into MNI space. All registrations were carried out in SPM8. After a nearest-neighbor interpolation, an 8 mm full-width at half-maximum Gaussian Kernel was then used to smooth the normalised lesion masks.

Two patients (one with SPMS, one with PPMS) were excluded from the subsequent imaging analysis due to inadequate registration.

### Co-localisation of GM atrophy and lesions

Voxel-based morphometry (VBM) and GM lesion probability mapping (LPM) analyses were carried out in SPM8. Two types of analyses were carried out: voxel-wise comparisons and region of interest (ROI) analyses. For voxel-wise VBM comparison between groups, we used a significance level of 0.05 (family wise error (FWE) corrected). For voxel-wise LPM, we used a significance level of 0.001 (uncorrected). This threshold was used as no lesion clustering was found at 0.05 (FWE corrected). In VBM and LPM comparisons, people with MS were compared to healthy controls to assess areas significantly more affected in people with MS.

To determine co-localisation of volume loss and DIR-visible GM lesions, permutation tests were run using Randomise^22^ implemented in FSL (FMRIB's software library, http://www.fmrib.ox.ac.uk/fsl). For each of the MS groups, the following regression model was tested per voxel (ie, with LPM as a voxel-wise covariate; sometimes known as biological parametric mapping^23^):
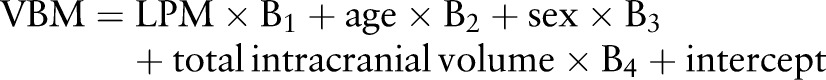


Total intracranial volume (ICV) was estimated by summing the thresholded GM, white matter (WM) and cerebrospinal fluid volumes using the ‘get_totals’ function in SPM8.

FSL Randomise uses a non-parametric permutation-testing procedure that derives an empirical null distribution without making assumptions about the normality of the data. The tests were performed in an inclusive GM mask (the average of the GM segments over subjects, thresholded at 0.5), using a cluster-forming threshold, and an uncorrected cluster-extent-based significance level of 0.01, with 5000 permutations.

**Figure 1:**
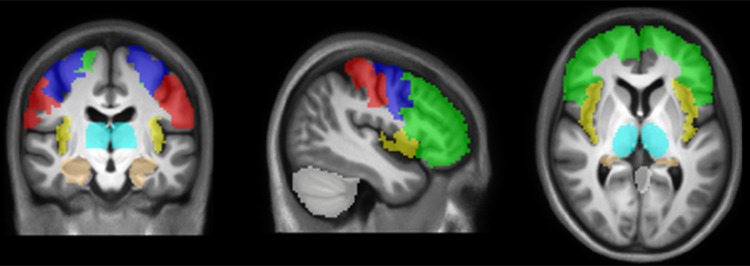
A priori defined regions of interest. A priori defined regions that were used in the present study: cerebellum, insula, precentral gyrus, postcentral gyrus, prefrontal cortex, medial temporal lobe and thalamus. Regions are overlaid on the cohort-specific MNI-space template (x=−43, y=3, z=−15).

The second analysis employed an ROI approach. ROIs involved in tasks assessing clinical and cognitive functioning in MS^24^ ^25^ were chosen a priori. These regions were the bilateral cerebellum, medial temporal lobe, postcentral gyrus, precentral gyrus, insula, prefrontal cortex and thalamus (figure 1). Masks for these regions were created by using Freesurfer to automatically segment the MNI-space cohort-specific T1-weighted template.^26^ ^27^ For each GM ROI and in each patient, the total GM lesion volume and the GM volume were extracted from the VBM and LPM images, and co-localisation between atrophy and lesion load was assessed using linear regression analyses, correcting for age, sex, and ICV.

### Associations of disability with GM lesion load and GM atrophy

To explore the associations of DIR-visible GM lesion load and GM atrophy with clinical measures (EDSS, PASAT, 9HPT, 25TWT, executive function and memory function), voxel-wise and ROI analyses were carried out. The first analysis, based on a voxel-wise factorial design, was conducted in SPM8, including age, sex and ICV, and using VBM or LPM values. In all voxel-wise comparisons, a cluster threshold of five voxels was applied.

Linear regression models used EDSS, PASAT, 9HPT, 25TWT, executive function and memory function as the dependent variables; GM lesion load and GM volume extracted from the ROIs were used as the independent variables. Age, sex and ICV were also added to the model as additional covariates. SPSS (V.21.0. Armonk, New York, USA: IBM Corp) was used to conduct this analysis. Differences between subtypes in scores, volume loss and lesion load were analysed using unpaired t tests (for VBM values) or non-parametric tests (for lesion-loads since they were non-normally distributed). When both GM lesion load and GM atrophy within a given ROI were associated with clinical performance, a linear regression model including lesion load, total GM volume, age and sex was run to determine the independent contribution of each to the outcome of interest. Shapiro-Wilk tests were used to assess normality of residuals of the linear regression analyses and unpaired t tests.

## Results

### Demographics and clinical performance

Thirty of the MS group had relapsing remitting MS (RRMS), 25 had PPMS, and 25 had SPMS. Demographics are shown in table 1.

**Table 1:** Demographics and clinical performance

	PPMS	RRMS	SPMS	All patients	Healthy controls
Females/males	14/11	20/10	14/11	48/32	18/12
Age (years)*	52.5 (9.8)	42.5 (9.6)	52.8 (7.6)	48.8 (10.2)	37.8 (11.8)
Disease duration (years)†	12.0 (7.4)	11.5 (10.5)	24.0 (8.2)	15.6 (10.5)	–
DMT (number of patients)	1	20	7	28	–
Pychotropic drugs (number of patients)	6	8	9	24	–
Benzodiazepines (number of patients)	3	0	0	3	–
**Median EDSS (range)**‡	6.0 (0.0–6.5)	1.75 (1.0–6.5)	6.5 (4.5–8.5)	5.75 (0.0–8.5)	–
HADS depression§	6.40 (3.59)	5.83 (3.56)	7.12 (3.69)	6.41 (0.40)	2.87 (3.50)
HADS anxiety	6.08 (4.33)	6.93 (3.31)	7.16 (4.18)	6.74 (0.44)	5.77 (4.41)
**z PASAT**	−0.70 (1.38)	−0.69 (1.32)	−0.94 (1.12)	−0.77 (1.27)	0.12 (1.05)
**z 9HPT**	−1.00 (1.13)	−0.66 (0.65)	−1.16 (0.90)	−0.93 (0.92)	0.63 (0.61)
**z 25TWT**	0.32 (0.97)	0.00 (1.03)	0.56 (1.01)	0.24 (1.02)	−0.44 (0.08)
Composite z MSFC	−0.62 (0.81)	−0.41 (0.76)	−0.77 (0.66)	−0.56 (0.76)	–
Hayling	4.80 (2.02)	5.17 (2.09)	4.64 (2.43)	4.89 (2.16)	6.17 (1.76)
Stroop	174.4 (76.7)	135.83 (36.9)	174.58 (80.9)	158.59 (66.7)	109.80 (20.0)
SDMT	43.44 (11.91)	52.33 (9.88)	41.09 (10.45)	46.23 (11.71	63.10 (9.91)
**Composite z executive**¶	−1.58 (1.69)	−0.71 (1.09)	−1.51 (1.55)	−1.23 (1.48)	0.23 (0.69)
Story recall immediate	30.04 (10.96)	33.97 (11.35)	27.76 (11.44)	30.80 (11.42)	37.13 (10.35)
Story recall delay	27.20 (12.29)	31.57 (10.93)	24.80 (12.22)	28.09 (11.97)	34.90 (10.31)
Figure recall immediate	51.09 (15.41)	62.20 (11.14)	51.57 (16.17)	55.80 (14.86)	67.67 (11.94)
Figure recall delay	48.73 (15.84)	60.07 (11.22)	48.67 (14.83)	53.37 (14.72)	66.30 (12.06)
RMT Words	46.17 (3.63)	47.60 (2.43)	45.61 (3.69)	46.56 (3.31)	49.00 (1.26)
RMT Faces	40.21 (5.27)	42.33 (4.23)	37.43 (5.53)	40.21 (5.31)	44.27 (3.20)
Composite z memory**	−1.30 (1.15)	−0.55 (0.89)	−1.63 (1.27)	−1.13 (1.18)	0.00 (0.58)

Mean values are presented and values in parentheses are SDs, unless specified otherwise. Clinical scores presented in bold are used in image analyses to study different domains of clinical function.

*Patients with RRMS were significantly younger than patients with PPMS and SPMS (both p<0.001).

†Patients with SPMS had longer disease duration than patients with RRMS and SPMS (both p<0.001).

‡All MS subgroups differed significantly (all p<0.05).

§All MS subgroups differed in their levels of depression from controls (all p<0.05), but not between each other.

¶Patients performed worse than healthy controls (p<0.01).

**Controls performed than patients (p<0.01). Patients with RRMS performance was better than patients with PPMS, who in turn performed better than the SPMS subgroup (p<0.05 and p<0.01, respectively).

9HPT, 9-hole Peg Test; MS, multiple sclerosis; PASAT, Paced Auditory Serial Addition Test; PPMS, progressive primary multiple sclerosis; RRMS, relapsing–remitting multiple sclerosis; SDMT, Symbol Digit Modalities Test; SPMS, secondary progressive multiple sclerosis; 25TWT, 25-foot Timed Walk Test.

All MS subgroups differed significantly in their EDSS scores (all p<0.05), with SPMS having a higher EDSS than patients with PPMS, who in turn had a higher EDSS than patients with RRMS (see table 1). All patient groups had significantly higher levels of depression than controls (all p<0.05), but no significant difference was found between subgroups. Anxiety levels were comparable between patients and controls. People with MS performed worse than healthy controls in all clinical domains. Patients had poorer executive functioning and memory function than healthy controls (all p<0.01). Memory functioning was best in patients with RRMS, followed by patients with PPMS, who in turn performed better than the SPMS subgroup (p<0.05 and p<0.01, respectively). When correcting for depression on clinical functioning, except for the subgroup comparison of patients with SPMS with PPMS on memory function, all group comparisons remained significant on all domains.

### GM atrophy

**Figure 2:**
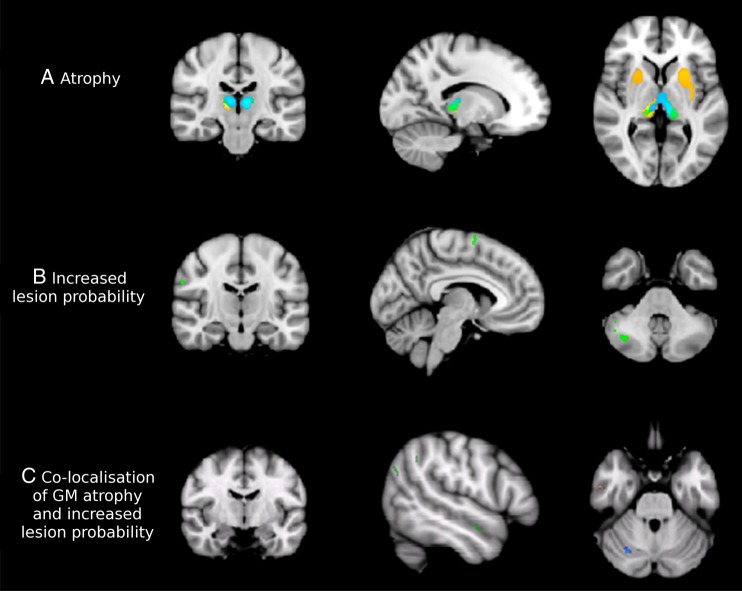
Grey matter (GM) pathology in MS. The progressive primary multiple sclerosis (PPMS) group is presented in green, relapsing–remitting multiple sclerosis (RRMS) in blue and secondary progressive multiple sclerosis (SPMS) in orange on the cohort-specific MNI-space template. (A) All multiple sclerosis (MS) subtypes show pronounced deep volume loss compared to controls at p<0.05 (family wise error, FWE corrected). In particular, patients with relapse onset have severe deep atrophy. Coordinates of sections are x=−14, y=−22, z=3. (B) Areas showing clusters of increased GM lesion probability in patients compared to controls at p<0.001, uncorrected. The RRMS group did not show clusters of increased GM lesion probability at this threshold, while SPMS showed increased cerebellar lesion load and PPMS had clusters of increased lesion probability throughout the cerebral and especially the cerebellar cortex. Coordinates of sections are x=−5, y=−13, z=−39. (C) Areas showing a significant correlation of atrophy and increased probability of a GM voxel being lesional (at p<0.01, uncorrected) in all patient groups together. Coordinates of sections are x=29, y=−66, z=−28.

People with MS showed significant GM atrophy compared to controls (figure 2A), predominantly in deep GM structures (thalamus, pallidum, putamen and caudate) in addition to a few small regions in the frontal (0.09 cm^3^), insular (0.06 cm^3^) and temporal lobes (0.12 cm^3^). Similarly, in the a priori defined ROIs (ie, cerebellum, medial temporal lobe, postcentral gyrus, precentral gyrus, insula, prefrontal cortex and thalamus, figure 1), mean GM volume was significantly smaller in patients than controls in the thalamus (p<0.001) and insula (p<0.05). Subgroup analyses, which showed that the most extensive atrophy in the deep GM regions and cortical areas was seen in SPMS, are reported in the online supplementary material.

### GM lesions

In patients, 1476 DIR visible lesions were found throughout the GM (neocortex: 1276, cerebellum: 154, deep GM: 46), with a mean total lesion volume of 1.20 cm^3^ (SD=0.92 cm^3^). Fourteen GM lesions were identified in healthy controls (in 4 participants).

At a threshold of p<0.001, patients had significantly higher GM lesion probability only in the right cerebellar hemisphere when compared with healthy controls (0.07 cm^3^, t_max_=3.37; figure 2B). No deep GM structures showed a significantly increased lesion probability at this threshold or at a lower threshold (p<0.01, uncorrected). However, at this lower threshold, additional cortical areas showed increased lesion probability, notably the right precentral and postcentral gyri, bilateral supplementary motor area, and bilateral temporal lobes (data not shown). People with MS had significantly more lesions in every ROI than healthy volunteers (all p<0.001). Subgroup analyses, which showed that PPMS and SPMS had regions of higher GM lesion probability than healthy controls, are detailed in the online supplementary material.

### Co-localisation of GM atrophy and lesions

In the whole MS group, voxel-wise analyses showed that there was little co-localisation of GM atrophy and lesions. Overall the volume of co-localisation was much smaller (less than 1 cm^3^) when compared with total brain GM volumes (>500 cm^3^). Areas that showed a significant association between a higher probability of a voxel being lesional and smaller GM volume were scattered throughout the brain, particularly in the cerebellum (figure 2C). ROI analyses found that increased GM lesion load was significantly associated with reduced GM volume within the cerebellum (B=−17.76, p<0.001) and postcentral gyrus (B=−4.77, p=0.037). The greatest extent of co-localisation was found in the PPMS cohort, while no co-localisation was seen in any group in the deep GM regions (see online supplementary material for more details on the subgroup analyses).

### Associations of GM atrophy and lesion load with clinical status

Voxel-wise models at p<0.05 (FWE corrected) in people with MS revealed an association between lower executive function scores and decreased left putamen volume (0.10 cm^3^), which was the only significant voxel-wise association of volume loss with any clinical metric. Furthermore, at p<0.001 (uncorrected), increased lesion probability in the cerebellum in particular was associated with lower performance in executive function, TWT speed and PASAT (table 2).

**Table 2:** Regions with increased lesion probability associated with poorer performance in clinical domains (thresholded at p<0.001 uncorrected)

Domain	Region	Side	cm^3^	Peak T value	MNI coordinates of local maxima		
Executive function	Precentral gyrus	R	0.08	3.75	48	8	29
	Inferior frontal operculum	R	0.24	3.84	49	9	28
	Anterior cingulum	L	0.07	3.48	−2	43	14
	Cerebellum crus1	R	0.18	3.51	40	−65	−37
	Cerebellum crus2	R	0.04	3.48	40	−65	−38
	Cerebellum 8	R	0.01	3.32	38	−57	−47
Memory function	Superior frontal lobe	R	0.01	3.29	17	3	72
25TWT	Cerebellum crus2	L	0.02	3.31	−30	−77	−36
zPASAT	Supramarginal gyrus	R	0.01	3.28	58	−39	33
	Cerebellum crus1	L	0.01	3.34	−29	−60	−38
	Cerebellum 6	L	0.03	3.59	−27	−58	−35
z9HPT	Precentral gyrus	R	0.04	3.36	11	−24	75

Lesion load in the cerebellum in particular is related to poorer executive, TWT speed, and PASAT performance.

9HPT, 9-hole Peg Test; zPASAT, Paced Auditory Serial Addition Test; 25TWT, 25-foot Timed Walk Test.

ROI analyses showed that cerebellar volume loss and increased GM lesion volume were associated with poorer functioning in all cognitive domains tested (table 3). In addition, lesions and volume loss within the postcentral gyrus were associated with cognitive performance and physical disability in almost all domains tested (ie, executive function, memory function, TWT speed, PASAT, 9HPT). Overall, lower functional scores were only weakly linked to GM lesion or atrophy clusters.

**Table 3:** MRI abnormality (volume loss and/or lesions) in ROIs significantly associated with poorer clinical function

Domain	ROI	Total GM volume				GM lesion volume			
		B	SE	p Value	Adj R^2^	B	SE	p Value	Adj R^2^
EDSS	Cerebellum	−0.053	0.022	0.018	0.203	**2.562**	**0.960**	**0.009**	**0.216**
	Postcentral gyrus	−**0.294**	**0.093**	**0.002**	**0.242**	5.441	2.326	0.022	0.199
Executive function	Cerebellum	**0.042**	**0.015**	**0.006**	**0.067**	−1.567	0.665	0.021	0.038
Memory function	Cerebellum	0.026	0.011	0.020	0.066	−**1.205**	**0.483**	**0.015**	**0.072**
	Postcentral gyrus					−2.695	1.164	0.023	0.062
z25TWT	Cerebellum	**0.014**	**0.006**	**0.020**	**0.156**	−0.543	0.244	0.029	0.148
	Postcentral gyrus	0.062	0.024	0.014	0.164	−**1.574**	**0.574**	**0.008**	**0.176**
zPASAT	Cerebellum	0.047	0.012	<0.001	0.150				
	Insula	0.336	0.099	0.001	0.122				
	Medial temporal lobe	0.286	0.094	0.003	0.098				
	Postcentral gyrus					−2.842	1.399	0.032	0.076
	Prefrontal lobe	0.026	0.011	0.025	0.052				
z9HPT	Cerebellum	0.023	0.009	0.016	0.100				

If both volume loss and increased lesion burden is associated, the strongest contributor is presented in bold.

EDSS, Expanded Disability Status Scale; GM, grey matter; z9HPT, 9-hole Peg Test; 25TWT, 25-foot Timed Walk Test; zPASAT, Paced Auditory Serial Addition Test, ROI, region of interest.

## Discussion

The present study assessed the distributions of DIR-visible GM lesions and GM atrophy in patients with MS, aiming to determine if they co-localise and if they both contribute to clinical outcomes. Co-localisation was at best modest, with the majority of atrophy occurring in regions that showed few GM lesions. Of the GM regions found to be consistently atrophic or to contain lesions, only the cerebellum and postcentral gyrus showed associations between both types of pathology, and physical and cognitive function.

Deep GM volume was reduced in patients compared to controls. This is in line with previous findings.^1^ ^28^ More limited cortical atrophy was also seen in regions consistent with past literature, including the right lateral prefrontal cortex,^29^ the left temporal and prefrontal cortex,^30^ and the bilateral superior and medial frontal gyrus and orbitofrontal regions.^31^ In addition, small areas of cortical atrophy were seen in the frontal, insular and temporal lobes, as reported previously.^5^ ^32^ The most extensive atrophy was seen in SPMS and a feature common to all subtypes was thalamic atrophy, which was particularly extensive in the SPMS group; this is in line with previous findings.^28^ ^33^

GM lesions were found throughout the cerebral and cerebellar cortex, particularly in the right cerebellum, but rarely in the deep GM structures. Co-localisation between GM atrophy and GM lesions was observed only when lower statistical thresholds were used, and then too mainly in the cortical regions. It was rarely seen in the deep structures, which showed atrophy. Work by Wegner *et al*^9^ has shown pathological changes within cortical lesions and to a lesser degree, in non-lesional cortex, but did not find an association between demyelination and cortical thickness, suggesting that demyelination per se is not directly responsible for cortical atrophy. Our findings are in agreement with this and we, therefore, suggest with caveats (see the discussion of the limitations of this work below) that GM atrophy and DIR-visible GM lesion are often not directly linked. This view is supported by recent findings that cortical MTR abnormalities, which is heavily influenced by demyelination, rarely co-localises with atrophy in MS,^20^ further suggesting differences in pathogenic mechanisms.

Previous papers have demonstrated that the topographic distribution of GM lesions is rather similar between patient subtypes, and that PPMS and RRMS share more similarities than differences in terms of GM lesion number, volume and topographic distribution.^5^ Additionally, there are differences in GM atrophy between subtypes of MS which involve not only a selective involvement of brain regions but also an increased extent of atrophy in common regions, such as the thalamus, in the progressive phase of MS.^34^ We confirmed these findings in this paper and therefore, combined all the phenotypes to investigate the overlap between lesions and atrophy. Based on our observed differences between the subgroups, we performed a sample size calculation to estimate the number of subjects a future study might require to detect (with 80% power at 5% signiﬁcance) a difference between subgroups in the volume of the prefrontal cortex (which was the ROI with the least power to detect differences) of 8 cm^3^ (as observed in our study), as well as in the proportion of lesional voxels of 0.22, using the means and SDs of concentrations provided by our data and standard methods for comparisons of means.^35^ We found that the sample size required to detect signiﬁcant GM volume differences between subtypes of MS was 75 per group for GM volume and 39 per group for GM lesion probability. This sample size calculation can be used by future studies that will aim to investigate the differences between groups in lesion probability and GM volume.

With regard to the association between GM lesions and atrophy with clinical disability, one interesting observation from the ROI analysis was that both GM lesion volume and atrophy in the postcentral gyrus (the primary somatosensory cortex) were linked to clinical measures of physical disability and to worse performance in almost all cognitive domains. Additionally, a significant and independent association was found between cerebellar lesion load and atrophy with lower physical and cognitive performance. This fits with the known involvement of the cerebellum in sensorimotor and cognitive function.^36^ In addition, the postcentral gyrus and cerebellum, which are both functionally and structurally connected,^36^ were the only regions in which lesion burden and volume loss were linked. Further work focusing on these two regions may shed light on the mechanisms of disability in MS. Using the voxel-wise analysis, we found that deep GM atrophy was generally not linked to functional outcomes, except for an association between executive function and a small region of atrophy in the putamen. This suggests that other processes, including white matter damage and brain network dysfunction, may be involved in cognitive dysfunction.^37^ ^38^

A number of limitations of this work should be taken into consideration. For LPM, some forms of cortical lesions may be easier to detect on DIR scans than others. DIR scans detect about a fifth of cortical lesions (18%) and less than a tenth of deep GM lesions (7%); also, while subpial lesions are the most abundant type seen in postmortem studies, these are rarely observed using DIR.^4^ Recent pathological work showed deep GM lesions and neurodegeneration in the deep GM that, in combination with cortical and white matter lesions, contributed to the clinical deficits;^39^ this insensitivity for DIR to detect deep GM lesions may partly explain the limited overlap found between lesions and atrophy in deeper GM structures. However, since the DIR-visible GM lesions represent the ‘peak’ of the real GM lesions, patients with higher DIR-visible lesions will also have higher number of real GM lesions; in this study we have investigated if these patients also have a greater amount of atrophy. Phase Sensitive Inversion Recovery MRI detects two to three times more GM lesions and so may increase the overall sensitivity of LPM analyses, but this sequence may still be less sensitive to subpial lesions than the other GM lesion subtypes.^40^ However, recent work has shown similar dissociations between the localisation of atrophy and demyelination, as measured by MTR,^20^ indicating that separations in the localisation and drivers of pathology are likely. A second limitation is that due to the low-frequency of overlapping lesions between patients we used a significance level of 0.001 (uncorrected) for the LPM analysis. While lowering the threshold increases the chance of a type I error, only one per 1000 voxels would show a false positive. In addition, the applied cluster threshold further reduces the number of false positive findings. Few studies have examined the effect of psychotropic drugs on cognition in MS. Oken *et al*^41^ found no significant effect of SSRIs and benzodiazepines on the PASAT, stroop, verbal memory or executive function test performance. These did, however, show an effect on a reaction time test. While use of psychotropic drugs may have subtly influenced our findings, the concomitant alleviation of depression, which is itself linked to cognitive impairment, is likely to have balanced out these effects. Not all patients were able to finish all clinical tasks due to disability, which led to the exclusion of their missing data from this task's analysis. It is, therefore, likely that the effects found were an underestimation of the real effects of MS as the excluded disabled patients were likely to also have the poorest cognitive function.

The present study assessed the distributions of DIR-visible GM lesions and GM atrophy in patients with different subtypes of MS and their relationship with clinical outcomes. We found that GM lesions (as seen using DIR) and GM atrophy do not usually co-localise, indicating that they are not directly spatially coupled. We also found that both GM lesions and atrophy separately contributed to disability, suggesting that the substrates of disability in MS are both pathologically and spatially heterogeneous.

## Supplementary Material

Web supplement

## References

[1] Geurts JJG, Calabrese M, Fisher E, et al Measurement and clinical effect of grey matter pathology in multiple sclerosis. Lancet Neurol 2012;11:1082–92. 10.1016/S1474-4422(12)70230-2 23153407

[2] Bø L, Vedeler CA, Nyland HI, et al Subpial demyelination in the cerebral cortex of multiple sclerosis patients. J Neuropathol Exp Neurol 2003;62:723–32. http://www.ncbi.nlm.nih.gov/pubmed/12901699 (accessed 30 May 2013). 12901699 10.1093/jnen/62.7.723

[3] Geurts JJG, Bø L, Pouwels PJW, et al Cortical lesions in multiple sclerosis: combined postmortem MR imaging and histopathology. AJNR Am J Neuroradiol 2005;26:572–7. http://www.ajnr.org/content/26/3/572.short (accessed 13 Feb 2013). 15760868 PMC7976495

[4] Seewann A, Kooi EJ, Roosendaal SD, et al Postmortem verification of MS cortical lesion detection with 3D DIR. Neurology 2012;78:302–8. 10.1212/WNL.0b013e31824528a0 22218278

[5] Calabrese M, Battaglini M, Giorgio A, et al Imaging distribution and frequency of cortical lesions in patients with multiple sclerosis. Neurology 2010;75:1234–40. 10.1212/WNL.0b013e3181f5d4da 20739644

[6] Calabrese M, Rocca MA, Atzori M, et al A 3-year magnetic resonance imaging study of cortical lesions in relapse-onset multiple sclerosis. Ann Neurol 2010;67:376–83. 20373349 10.1002/ana.21906

[7] Sepulcre J, Goñi J, Masdeu JC, et al Contribution of white matter lesions to gray matter atrophy in multiple sclerosis: evidence from voxel-based analysis of T1 lesions in the visual pathway. Arch Neurol 2009;66:173–9. 10.1001/archneurol.2008.562 19204153

[8] Mühlau M, Buck D, Förschler A, et al White-matter lesions drive deep gray-matter atrophy in early multiple sclerosis: support from structural MRI. Mult Scler 2013;19:1485–92. 10.1177/1352458513478673 23462349

[9] Wegner C, Esiri MM, Chance SA, et al Neocortical neuronal, synaptic, and glial loss in multiple sclerosis. Neurology 2006;67:960–7. 10.1212/01.wnl.0000237551.26858.39 17000961

[10] Polman CH, Reingold SC, Banwell B, et al Diagnostic criteria for multiple sclerosis: 2010 revisions to the McDonald criteria. Ann Neurol 2011;69:292–302. 10.1002/ana.22366 21387374 PMC3084507

[11] Kurtzke JF Rating neurologic impairment in multiple sclerosis: an expanded disability status scale (EDSS). Neurology 1983;33:1444–52. http://www.ncbi.nlm.nih.gov/pubmed/6685237 (accessed 22 May 2013). 10.1212/WNL.33.11.1444 6685237

[12] Zigmond AS, Snaith RP The hospital anxiety and depression scale. Acta Psychiatr Scand 1983;67:361–70. http://www.ncbi.nlm.nih.gov/pubmed/6880820 (accessed 11 Jul 2014). 10.1111/j.1600-0447.1983.tb09716.x 6880820

[13] Burgess P, Shallice T The Hayling and Brixton tests. Test manual. Bury St Edmunds, UK: Thames Valley Test Company, 1997.

[14] Trennery MR Stroop neuropsychological screening test manual. Odessa, FL: Psychological Assessment Resources, 1989.

[15] Smith A Symbol digit modalities test (SDMT): manual (revised). Los Angeles: Western Psychological Services, 1982.

[16] Coughlan A, Hollows S The adult memory and information processing battery (AMIPB). Leeds, UK: St James University Hospital, 1985.

[17] Warrington EK Manual for recognition memory test. Windsor, UK: NFER-Nelson, 1984.

[18] Chard DT, Jackson JS, Miller DH, et al Reducing the impact of white matter lesions on automated measures of brain gray and white matter volumes. J Magn Reson Imaging 2010;32:223–8. 10.1002/jmri.22214 20575080

[19] Ashburner J A fast diffeomorphic image registration algorithm. Neuroimage 2007;38:95–113. 10.1016/j.neuroimage.2007.07.007 17761438

[20] Mallik S, Muhlert N, Samson RS, et al Regional patterns of grey matter atrophy and magnetisation transfer ratio abnormalities in multiple sclerosis clinical subgroups: a voxel-based analysis study. Mult Scler 2015;21:423–32. 10.1177/1352458514546513 25145689 PMC4390521

[21] Geurts JJG, Roosendaal SD, Calabrese M, et al Consensus recommendations for MS cortical lesion scoring using double inversion recovery MRI. Neurology 2011;76:418–24. 10.1212/WNL.0b013e31820a0cc4 21209373

[22] Winkler AM, Ridgway GR, Webster MA, et al Permutation inference for the general linear model. Neuroimage 2014;92:381–97. 10.1016/j.neuroimage.2014.01.060 24530839 PMC4010955

[23] Casanova R, Srikanth R, Baer A, et al Biological parametric mapping: a statistical toolbox for multimodality brain image analysis. Neuroimage 2007;34:137–43. 10.1016/j.neuroimage.2006.09.011 17070709 PMC1994117

[24] Genova HM, Hillary FG, Wylie G, et al Examination of processing speed deficits in multiple sclerosis using functional magnetic resonance imaging. J Int Neuropsychol Soc 2009;15:383–93. 10.1017/S1355617709090535 19402924

[25] Cardinal KS, Wilson SM, Giesser BS, et al A longitudinal fMRI study of the paced auditory serial addition task. Mult Scler 2008;14:465–71. 10.1177/1352458507084263 18208900

[26] Fischl B, Salat DH, Busa E, et al Whole brain segmentation: automated labeling of neuroanatomical structures in the human brain. Neuron 2002;33:341–55.http://www.ncbi.nlm.nih.gov/pubmed/11832223 (accessed 22 May 2013). 10.1016/S0896-6273(02)00569-X 11832223

[27] Desikan RS, Ségonne F, Fischl B, et al An automated labeling system for subdividing the human cerebral cortex on MRI scans into gyral based regions of interest. Neuroimage 2006;31:968–80. 10.1016/j.neuroimage.2006.01.021 16530430

[28] Ceccarelli A, Rocca MA, Pagani E, et al A voxel-based morphometry study of grey matter loss in MS patients with different clinical phenotypes. Neuroimage 2008;42:315–22. 10.1016/j.neuroimage.2008.04.173 18501636

[29] Audoin B, Davies GR, Finisku L, et al Localization of grey matter atrophy in early RRMS: a longitudinal study. J Neurol 2006;253:1495–501. 10.1007/s00415-006-0264-2 17093899

[30] Morgen K, Sammer G, Courtney SM, et al Evidence for a direct association between cortical atrophy and cognitive impairment in relapsing-remitting MS. Neuroimage 2006;30:891–8. 10.1016/j.neuroimage.2005.10.032 16360321

[31] Sailer M, Fischl B, Salat DH, et al Focal thinning of the cerebral cortex in multiple sclerosis. Brain 2003;126:1734–44. 10.1093/brain/awg175 12805100

[32] Calabrese M, Atzori M, Bernardi V, et al Cortical atrophy is relevant in multiple sclerosis at clinical onset. J Neurol 2007;254:1212–20. 10.1007/s00415-006-0503-6 17361339

[33] Sepulcre J, Sastre-Garriga J, Cercignani M, et al Regional gray matter atrophy in early primary progressive multiple sclerosis: a voxel-based morphometry study. Arch Neurol 2006;63:1175–80. 10.1001/archneur.63.8.1175 16908748

[34] Ceccarelli A, Rocca MA, Falini A, et al Normal-appearing white and grey matter damage in MS. A volumetric and diffusion tensor MRI study at 3.0 Tesla. J Neurol 2007;254:513–18. 10.1007/s00415-006-0408-4 17401516

[35] Armitage P, Berry G, Matthews J Statistical methods in medical research. 4th ed. Blackwell Science Ltd, 2002.

[36] Stoodley CJ, Valera EM, Schmahmann JD Functional topography of the cerebellum for motor and cognitive tasks: an fMRI study. Neuroimage 2012;59:1560–70. 10.1016/j.neuroimage.2011.08.065 21907811 PMC3230671

[37] Muhlert N, Atzori M, De Vita E, et al Memory in multiple sclerosis is linked to glutamate concentration in grey matter regions. J Neurol Neurosurg Psychiatry 2014;85:833–9. 10.1136/jnnp-2013-306662 24431465 PMC4112488

[38] Muhlert N, Sethi V, Cipolotti L, et al The grey matter correlates of impaired decision-making in multiple sclerosis. J Neurol Neurosurg Psychiatry 2014;86:530–6. 10.1136/jnnp-2014-308169 25006208 PMC4413680

[39] Haider L, Simeonidou C, Steinberger G, et al Multiple sclerosis deep grey matter: the relation between demyelination, neurodegeneration, inflammation and iron. J Neurol Neurosurg Psychiatry 2014;85:1386–95. 10.1136/jnnp-2014-307712 24899728 PMC4251183

[40] Sethi V, Yousry TA, Muhlert N, et al Improved detection of cortical MS lesions with phase-sensitive inversion recovery MRI. J Neurol Neurosurg Psychiatry 2012;83:877–82. 10.1136/jnnp-2012-303023 22807559

[41] Oken BS, Flegal K, Zajdel D, et al Cognition and fatigue in multiple sclerosis: potential effects of medications with central nervous system activity. J Rehabil Res Dev 2006;43:83–90. http://www.ncbi.nlm.nih.gov/pubmed/16847774 (accessed 19 Mar 2015). 10.1682/JRRD.2004.11.0148 16847774

