# Supplementary material for: DIR-visible grey matter lesions and atrophy in multiple sclerosis: partners in crime?
Source: J Neurol Neurosurg Psychiatry. 2015 Apr 29;87(5):461–7. doi: 10.1136/jnnp-2014-310142 (PMC4853554; doi:10.1136/jnnp-2014-310142)
Supplement: Web supplement [file jnnp-2014-310142-s1.pdf]

SUPPLEMENTAL RESULTS

Demographics and clinical performance

SPMS patients had a longer disease duration than PPMS and RRMS patients (both p<0.001). EDSS scores differed significantly between MS subgroups (p<0.001), with the SPMS group having a higher EDSS than PPMS group, and people with PPMS having higher EDSS scores than those with RRMS (both p<0.05). SPMS patients had worse executive function than PPMS, and PPMS worse than RRMS (p<0.05 and p<0.01, respectively).

GM atrophy

Subgroup analyses revealed that deep GM volume loss was present in all MS groups compared to controls, with additional temporal lobe volume loss in the PPMS group, and volume loss of GM in the occipital lobe, amygdala and hippocampus in the SPMS group (**Supplemental table 1 and Figure 2a**). Voxel-wise comparisons between patient groups yielded no significant results, though ROI analyses revealed significant subgroup differences in insular and thalamic volumes. SPMS patients had a significantly reduced insular volume compared to both healthy controls and PPMS patients (both p<0.01, Bonferroni corrected for multiple comparisons). Thalamic volume was also reduced in SPMS compared to both healthy controls and PPMS patients (p<0.001 and p<0.05, respectively).

GM lesions

Mean GM lesion volume was 1.33cm^3 (SD = 1.04cm^3) for PP patients, 1.39 cm^3 (0.85) for SPMS patients, and 0.93cm^3 (0.82) for RRMS patients.

Group wise whole brain analyses at p<0.001 (**figure 2b**) revealed that PPMS patients had a significantly higher lesion probability than controls in the left supplementary motor area (0.35cm^3), right medial frontal lobe (0.05cm^3), right postcentral gyrus (0.03cm^3), right supra-marginal gyrus (0.09cm^3), and the right cerebellum (0.38cm^3). The RRMS subgroup did not show any significant lesional clusters compared to controls. The SPMS group showed consistent clustering of lesions in the supplementary motor area (0.05cm^3) compared to controls. All MS groups had significantly more lesions in every ROI than healthy volunteers

(all $p<0.001$), and were comparable among MS groups for all ROIs, with the exception of the cerebellum, in which those with SPMS showed more lesions than those with RRMS ($p<0.05$, Bonferroni corrected).

Co-localisation of GM atrophy and lesions

When we examined the co-localisation between volume loss and lesion load across MS subtypes (**supplemental table 2 and Figure 2c**), the PPMS group showed spatial overlap between the two pathological abnormalities throughout the cerebellar and cerebral cortex. While RRMS patients showed co-localisation of these forms of pathology mainly in the cerebellum, SPMS patients showed very little co-localisation, mainly in the post-central gyrus. The least co-localisation was found in the SPMS patients (0.01cm^3), whilst the RRMS patients (0.18cm^3) were in between the PPMS (0.30cm^3) and SPMS groups in terms of number of regions showing both forms of MR changes. None of the groups showed co-localisation in the deep GM (where significant atrophy was found in the absence of GM lesions). ROI-wise regression analyses in the RRMS group showed a significant association between cerebellar lesion load and volume loss ($B=-33.46$, $p<0.001$).

Supplemental table 1. Clusters of atrophic voxels in different MS subtypes.

At p<0.05 FWE corrected, all MS subtypes show deep volume loss (caudate, pallidum, putamen and thalamus) when compared with healthy controls. Particularly patients with SPMS have consistent deep atrophy.

MS subtype	Region	Side	cm^3	Peak T-value	MNI coordinates of local maxima		
					x	y	z
PP	Hippocampus	L	0.02	5.82	-13	-34	2
	Lingual	L	0.01	5.77	-13	-33	0
	Postcentral	L	0.01	5.45	-52	-13	31
	Putamen	L	0.05	5.49	-33	-10	-1
	Thalamus	L	0.40	6.37	-15	-29	2
	Thalamus	R	0.28	5.75	15	-27	5
	Temporal superior	L	0.09	5.78	-60	-28	14
RR	Caudate	R	0.15	5.58	13	6	20
	Thalamus	L	0.19	7.37	-10	-26	6
	Thalamus	R	0.15	6.59	10	-22	9
SP	Hippocampus	R	0.03	6.06	29	-9	-10
	Amygdala	R	0.10	6.30	31	-7	-10
	Occipital inferior	R	0.01	5.36	31	-97	-4
	Caudate	R	0.01	5.29	14	8	19
	Putamen	L	4.21	6.55	-26	6	-5
	Putamen	R	3.02	7.04	33	-6	-6
	Pallidum	L	0.47	6.35	-25	2	-3
	Pallidum	R	0.21	6.53	30	-9	-5
	Thalamus	L	1.37	8.99	-14	-25	5
	Thalamus	R	1.62	8.04	14	-25	5

Supplemental table 2. Areas in patients showing a significant association between smaller GM volume and increased lesion load.

At p<0.01 uncorrected, co-localisation of both forms of pathology was largest in patients with PPMS, throughout virtually the entire brain with the exception of deep GM. Particularly the cerebellum in RRMS patients showed a great association between these forms of pathology. Overall, lesion-atrophy clusters were small when compared with the volume of the brain.

MS subtype	Region	Side	cm^3	Peak T-value	MNI coordinates of local maxima		
					x	y	z
PP	Precentral gyrus	L	0.05	3.13	-32	-9	68
	Frontal superior	R	0.01	2.64	23	63	-5
	Hippocampus	L	0.01	3.98	-35	-19	-16
	Occipital superior	L	0.03	4.24	25	-74	24
	Occipital middle	L	0.01	2.93	-26	-74	24
	Parietal inferior	L	0.07	3.14	-50	-69	30
	Temporal superior	R	0.01	2.84	53	-18	-6
	Temporal middle	L	0.08	3.91	-50	-6	-15
	Cerebellum	R	0.03	5.11	19	-66	-28
RR	Temporal superior	R	0.01	4.16	60	-19	-5
	Cerebellum	R	0.17	3.62	29	-68	-29
SP	Postcentral gyrus	R	0.01	2.51	14	-42	62
